# Effectiveness of a worksite lifestyle intervention to reduce BMI among farmworkers in California: a cluster randomised controlled trial

**DOI:** 10.1017/S136898002200129X

**Published:** 2022-09

**Authors:** Susana L Matias, Heather E Riden, Deandra S Lee, Heejung Bang, Marc B Schenker

**Affiliations:** 1 Nutritional Sciences and Toxicology, University of California, 225 Morgan Hall, Berkeley, CA 94720, USA; 2 Public Health Sciences, University of California, Davis, CA, USA; 3 Western Center for Agricultural Health and Safety, University of California, Davis, CA, USA; 4 Center for Health and Environment, University of California, Davis, CA, USA

**Keywords:** Farmworkers, Latinos, Obesity, Workplace intervention, Lifestyle intervention, California

## Abstract

**Objective:**

To evaluate the effectiveness of *PASOS SALUDABLES*, a culturally tailored lifestyle intervention to prevent obesity and diabetes among Latino farmworkers, when implemented at large scale in the worksite.

**Design:**

This study was a two-arm parallel group, cluster randomised controlled trial, where participants received either a twelve-session lifestyle intervention (intervention) or six-session leadership training (control) at their worksite. The intervention was delivered by *Promotoras* in Spanish. All sessions were conducted at the worksites (ranches) during meal breaks. Blinded, trained research assistants collected socio-demographic and outcome data (i.e. BMI as primary outcome and waist circumference, glycated Hb (HbA1c), cholesterol and blood pressure as secondary outcomes) at baseline and follow-up assessments (i.e. 3 months, 6 months, 1 year and 1·5 years).

**Setting:**

Recruitment and intervention delivery occurred at twelve study ranches in Oxnard, California.

**Participants:**

We enrolled farmworkers hired by a large berry grower company, who were ≥18 years old, spoke Spanish and were free of diabetes at screening.

**Results:**

A total of 344 workers were enrolled in the intervention and 271 in the control group. The intervention resulted in attenuated increase of BMI over time; however, the difference in trend between groups was not significant (*β* = −0·01 for slope difference, *P* = 0·29). No significantly different trend by group was observed in secondary outcomes (*P* > 0·27).

**Conclusions:**

The worksite intervention, implemented during meal breaks, did not reduce BMI or other clinical indicators. Nevertheless, this study supports the feasibility of recruiting and engaging the Latino farmworker population in workplace health promotion interventions.

California produces more than half of the fruits and nuts (54 %) and vegetables (61 %) consumed in USA households^(1)^. These labour-intensive crops employ more than 800 000 farmworkers, most of whom are Latino immigrants, and about 60 % have no work authorisation^(2)^. California’s farmworker population is mostly male (73 %), foreign-born (87 %) and low educated (26 % completed high school)^(3)^. In 2013–2014, 29 % of agricultural workers had a total family income below the federal poverty level, and only one-third had health insurance^(3)^. They also face much higher rates of food insecurity (45 %–66 %) compared with the general USA population^(4–6)^. Not surprisingly, their burden of chronic disease is also particularly high: 79 %–94 % had overweight or obesity^(7–9)^, 15 % had diabetes and 26 % had hypertension^(7)^.

Despite the high risk of chronic conditions, lifestyle interventions targeting Latino farmworkers are lacking. Research evidence suggests that lifestyle interventions targeting diet modification and physical activity result in weight loss (5·6 kg) in the general population^(10)^. However, only a few community-based lifestyle interventions have been developed targeting Latino populations^(11–13)^. Furthermore, to our knowledge, no worksite-based approach has been implemented with Latinos in general or in an agricultural setting. To fill this gap, and in collaboration with a large berry producer in the USA, we developed and pilot tested *PASOS SALUDABLES*, a 10-week culturally relevant healthy lifestyle worksite intervention developed by UC Davis to prevent diabetes and reduce obesity in Latino farmworkers^(14)^. The pilot study included 254 farmworkers and demonstrated the feasibility of a workplace intervention (delivered at employer-run health clinics), as a promising venue for reaching a population that spends considerable time working in the fields. Using a randomised controlled trial design, we demonstrated modest but statistically significant reductions in BMI and waist circumference particularly among females, and positive changes in dietary and physical activity behaviours in the intervention group^(14)^. Since such lifestyle behaviour changes can reduce worksite absenteeism^(15)^, and worksite obesity interventions return-on-investment include more productivity and reduced medical costs^(16,17)^, further evaluation of this intervention in the agricultural workforce was justified.

The current study was conducted to expand our previous pilot work by implementing and evaluating the effectiveness of *PASOS SALUDABLES*, a lifestyle intervention delivered at the workers’ agricultural worksite fields, implemented on a larger scale (i.e. with a larger sample size in a less controlled environment) and with a longer follow-up period to inform sustainability of effects. Specifically, we aimed to assess changes due to the intervention in the primary (i.e. BMI) and secondary (i.e. waist circumference, glycated Hb (HbA1c), cholesterol and blood pressure) clinical outcomes.

## Methods

### Study design

The *PASOS SALUDABLES* study was a cluster randomised controlled trial where clusters were allocated in a 1:1 ratio of intervention to control in parallel groups (ClinicalTrials.gov Identifier NCT02480244). Eligible clusters were defined as the ranches where approximately 100 farmworkers worked; random allocation occurred at the ranch level. Study ranches were selected based on the number of crews and workers, their geographic location, crop (i.e. berry type), prior participation in another lifestyle employer-run worksite program (i.e. *Sembrando Salud*), approval from employer leadership and interest from ranch managers. The employer sent ranch information to the research team, and the study statistician conducted the randomisation. Whenever two ranches were identified as eligible, the study statistician randomly allocated one to the intervention group and one to the control group. Ranches were selected in pairs to account for staffing needed to enroll participants, collect data and deliver sessions in the same timeframe.

Ranches allocated to the intervention group received the twelve-session intervention over 6–12 weeks, while ranches allocated to the control group received no intervention, but were offered a six-session leadership training over 3–6 weeks.

Midway through the recruitment phase, the employer changed its approach to labour management. Ranch managers began to oversee multiple ranches within reasonable proximity of each other, and although work crews remained in the same commodity, they were rotated between ranches under the same manager. In some cases, crews were temporarily moved into study ranches. Attempts to mitigate the impact of this labour management change included close communication between a point person staff for the study and the ranch managers.

### Participants

After randomisation, *Promotoras* or community health leaders, hired and trained by the research team visited intervention and control ranches to recruit participants. Interested workers provided their contact information and research assistants followed up by phone to set up an appointment to administer a screening questionnaire over the phone or in-person and determine their eligibility to participate in the study.

At the individual level, the following inclusion criteria were used: (1) work at Reiter Brothers, Inc., a partner or affiliate company; (2) be at least 18 years of age; (3) plan to stay in the area for the next 3 months; (4) be willing to attend weekly sessions for 6–12 weeks and (5) be able to speak and read Spanish. Exclusion criteria included (1) workers who could not communicate in Spanish; (2) pregnant women, or women planning a pregnancy within 6 months or breast-feeding (unless discontinuing within 1 month); (3) individuals unable to undertake moderate physical exercise, taking medicine for high blood pressure or heart conditions, having bone or joint problems, loss of consciousness or falls due to dizziness or having developed chest pain within the last month; (4) individuals taking medications that affect weight or following therapeutic diets; (5) previous diabetes diagnosis, or HbA1c ≥ 6·5 % at screening; (6) individuals with a spouse/cohabitant already enrolled in the study and (7) individuals who have previously participated in the employer’s lifestyle intervention *Sembrando Salud* within the last 4 years (this was changed to 2 years after the trial started, to increase eligibility rate).

Individuals determined to be eligible to participate in the study were invited to schedule an in-person meeting with a research assistant to review and sign the informed consent document and complete the baseline clinical data collection and questionnaire.

The study protocol with details on study design, including sample size calculations has been published^(18)^. Briefly, under the assumption of independence, the minimal total sample required for detecting a 4 % difference in weight was 52 (twenty-six per group) and for detecting a 3 % difference was 90 (forty-five per group) with 80 % power and alpha level of 5 % using two-sided hypothesis testing. These minimum detectable effect sizes were based on previously reported effect sizes from a community-based lifestyle intervention^(19)^. To take into account the cluster design, we used an inflation factor, defined as inflation factor = [1 + (m-1)*ρ], where m is the average cluster size and ρ is the intra-class correlation. Assuming an intra-class correlation equal to 0·02, the minimal total sample increased to 259 (4 % difference) and 449 (3 % difference); considering the average cluster (ranch) size (∼100 workers), it was estimated that a minimum of 4–6 clusters were needed.

### Study setting

The study was conducted in Oxnard, California. Recruitment and intervention occurred at the study ranches. *Promotoras* visited the selected ranches to share information about the study and invite individual work crews to participate. Recruitment at ranches was staggered, so that as soon as there were enough interested participants and once baseline assessments were completed, the educational sessions started at the designated ranches. All data collection, including clinical measures, occurred at a centralised employer’s office to facilitate the collection of the clinical measurements at a more efficient rate. All educational sessions were conducted at the study ranches. Some supplemental activities were held in the employer’s office or in the community (e.g. library and community centre).

### Intervention

*PASOS SALUDABLES* was a worksite lifestyle intervention to prevent obesity and diabetes. The *PASOS* intervention consisted of a culturally tailored curriculum appropriate for delivery in an agricultural setting. It was designed to educate participants about obesity, diabetes and healthy lifestyles, motivate behavioural changes in diet and exercise habits and provide a supportive participatory group setting. The content was based on the *Salud para su Corazon* program, developed by the U.S. National Heart, Lung and Blood Institute^(20)^ and the *5 Pasos* (five steps in English) social media campaign, implemented in Mexico by the Mexican Government^(21)^. The *Salud para su Corazon* curriculum is a user-friendly, bilingual programme for *Promotoras* specifically developed for Latino communities; it was used to supplement some of the visual aids used during the intervention delivery. The five steps were (1) Move; (2) Drink water; (3) Eat fruits and vegetables; (4) Measure (food portions and weight) and (5) Share (information learned and healthy habits).

The core intervention programme consists of twelve lessons, and it was delivered over 6–12 weeks. To maximise participation and retention in this workforce, which averages long work hours, the core intervention sessions took place at the ranches during work hours while farmworkers were on a meal break. Because of this, the 90-min sessions developed and tested previously^(14)^ had to be shorten considerably to take approximately 20 min, which was achieved by removing the ∼20 min for physical activity during the sessions and shortening content. Sessions were presented to work crews in a format analogous to tailgate trainings. Tailgate trainings are gatherings of small groups of workers around the tailgate of a truck, in the field, or elsewhere for a brief, informal and focused training session on a single topic. The intervention was delivered by *Promotoras* who were extensively trained on curriculum content, framework of the intervention, group management and effective presentation delivery skills, to ensure that participants understood the material and were able to motivate and support participants. In addition, a few supplemental activities (i.e. workshops) were offered throughout the duration of the study and included topics such as a nutrition labelling, diabetes awareness and stress management.

Participants in the control group received six educational sessions over an average of 6 weeks. The control sessions utilised the employer’s leadership training material for farmworkers on empathy, communication, conflict resolution and sharing knowledge. Sessions for control participants were also held at the ranch during a meal break. Recruitment and follow-up for control participants followed a similar approach as those for intervention participants.

### Outcome measures

The primary outcome measure was BMI, calculated as kg/m^2^. Weight was measured in kilograms using an EatSmart Precision Digital Bathroom Scale (Health Tools, LLC.), with participants dressed in light clothing and without shoes. Standing height was measured in centimetres with a Seca 213 mobile stadiometer (SECA, Chino).

Secondary clinical outcomes included clinical measures of glycated Hb (HbA1c; %), total cholesterol (mg/dl), blood pressure (mm Hg) and waist circumference (cm). HbA1c was measured with the DCA Vantage™ (Siemens Medical Diagnostic Solutions), and a point-of-care immunoassay analyser that measures the percent concentration of HbA1c in blood. Total cholesterol was analysed using the Cholestech LDX® System (Cholestech Corporation). Both of these point-of-care testing devices have been utilised in population-based community settings and produced accurate and reproducible results, when compared with ‘gold standard’ laboratory measures^(22,23)^. Blood pressure was measured in standard fashion, using an automated device that employs standardised Doppler procedures, following procedures developed by the American Heart Association^(24)^. Systolic and diastolic blood pressure measures were recorded. Waist circumference was measured using a Gulick II tape measure (Model 67020).

Behavioural and lifestyle secondary outcomes such as dietary and physical activity patterns were also assessed. Findings on those outcomes will be reported separately.

### Data collection

Data were collected at five different points: (1) baseline (visit 0); (2) approximately 3-month follow-up (at the end of intervention; visit 1); (3) 6-month follow-up (visit 2); (4) 1-year follow-up (visit 3) and (5) 1·5-year follow-up (visit 4).

Anthropometry (i.e. weight, height and waist circumference) and systolic and diastolic blood pressure were assessed at each data collection point. Clinical (i.e. HbA1c and total cholesterol) and behavioural lifestyle (i.e. nutrition and physical activity) outcomes were assessed at baseline, the 3-month follow-up and the 1-year follow-up. Information about socio-demographics, acculturation, medical history, smoking habits, alcohol consumption, health perception and knowledge of dietary and physical activity recommendations was also collected at those time points, using an interviewer-administered questionnaire.

Two trained research assistants collected all study data, including the clinical measures. Attempts were made to keep the research assistants blind to group allocation throughout the data collection process. During the baseline clinical testing, individuals who had an HbA1c of ≥ 6·5 % were tested a second time. If both tests were ≥ 6·5 % (*n* 13), they were informed that they were not eligible, referred for medical care (to the employer’s clinic) and withdrawn from the study. In the case of a missed 3-month or 1-year follow-up, participants were asked to complete the full questionnaire, instead of just the clinical measures, at the 6-month or 1·5-year follow-up, respectively, to obtain more complete data.

Participants were offered incentives to participate in the longitudinal data collection sessions. Initially, participants received $20–$25 gift cards, which were later increased to $50–$75 gift cards to enhance recruitment and retention. Participants who enrolled in the study prior to the increased incentive were provided with the difference at their next data collection interview. An additional $100 incentive was given to individuals who completed all five data collection visits.

### Statistical analysis

For the primary analysis, we followed a modified intent-to-treat approach, that is, excluding participants who did not attend a minimum of three intervention sessions and with no imputation of outcome data for losses to follow-up. The statistician was blind to treatment allocation during data collection and analyses, e.g. analysing the data with A *v*. B coding. Participant characteristics at baseline were summarised using standard descriptive statistics, i.e. mean (SD) for continuous variables and frequency (proportion) for categorical variables, by intervention status. Next, we drew time plots for primary and secondary outcomes (all continuous variables) with sample (unadjusted) means at each time point along with pointwise 95 % CI, using visit number (e.g. baseline = visit 0, 3-month follow-up = visit 1, etc.) as the time variable for clear visualisation with common time configuration. In the statistical analyses, actual follow-up month, which can vary for different participants, was used.

For primary hypothesis testing, we used mixed effects models for longitudinal data^(25)^ to account for within-participant correlation. This was decided based on a very low within-cluster/ranch correlation for the primary outcome (intra-class correlation ≈ 0·00). Models included the treatment indicator variable (intervention *v*. control), the time variable (i.e. follow-up month, treated as continuous variable) and the interaction of these two variables.

In longitudinal (repeated measures) data analysis, the primary parameter is the regression (*β*) coefficient for the interaction of the treatment and time, which captures the slope difference (or time trajectory/trend) of the outcome variable between the two groups, while the regression coefficients for treatment indicator and time capture imbalance in the outcome (e.g. BMI) at baseline and time trend of the outcome for the reference (control) group, respectively.

We also conducted sensitivity analyses by fitting these additional models: (a) with three-level nested clustering (outcomes within person within ranch); (b) visit number as a categorical variable, which can capture nonlinearity of time trend; (c) excluding participants with HbA1C ≥ 6·5 % at baseline and (d) gender adjustment, based on the somewhat different distribution between the two arms (which can happen in cluster randomised controlled trial with small number of clusters). We did not conduct interim analyses based on the study protocol. SAS 9.4 (SAS Institute, Inc.) was used for data analyses.

## Results

### Recruitment and retention

**Fig. 1:**
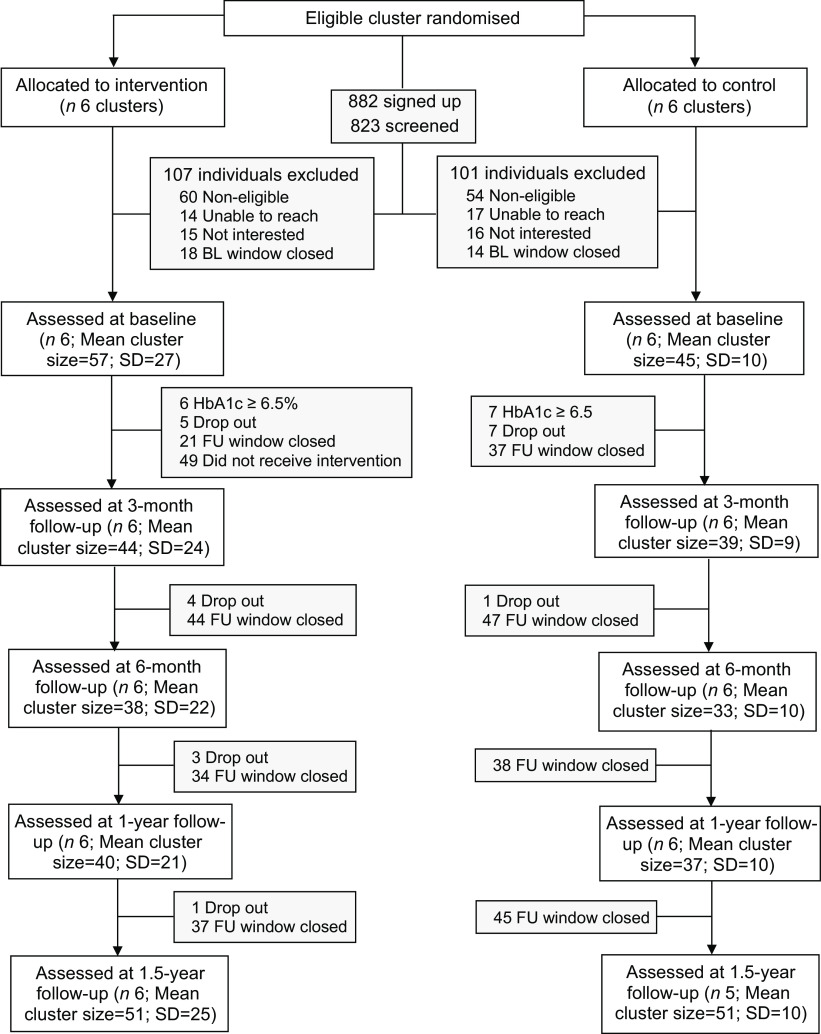
Light grey colour filled boxes list sample sizes at the individual level. ‘Window closed’ indicates that the time window for completing that visit was over. ‘Drop out’ refers to a participant who was no longer in the study (no longer interested or employed, lack of transportation, out of the country, moved, passed away, and withdrew due to misconduct). ‘Unable to reach’ means a participant could not be reached after a predefined number of attempts to contact them. BL, baseline; HbA1c, glycated Hb; FU, follow-up; sd, standard deviation of cluster size; *n* refers to sample size at the cluster level

Recruitment began in August 2015 and was completed in August 2017. Twelve ranches were randomised in the study (six to the intervention group and six to the control group), and a total of 1366 workers were presented with the opportunity to enroll. Approximately 65 % (*n* 882) of the workers who were informed about the study were screened. Of those screened, 83 % (*n* 735) were eligible to participate in the study and 70 % (*n* 615) enrolled and completed the baseline assessment (Fig. 1). Follow-up assessments were conducted between November 2015 and December 2018.

A total of 344 workers were enrolled in arm 1 (intervention) and 271 in arm 2 (control). Visual inspection (e.g. without formal comparison following CONSORT guidelines for Table 1 in controlled trials) revealed no notable imbalance in baseline characteristics between arms. However, due to cluster randomisation with a relatively small number of clusters, some statistical imbalances (including sample size) were unavoidable. Mean age was ∼34·5 years old (sd = 9·4) in both arms, but there was a slightly higher proportion of women in arm 2 (49 *v*. 46 %). Over 95 % of participants were from Mexico and over 74 % spoke Spanish as their primary language. With regard to crops, raspberry was the most common berry type, and harvester was the most common job type. Medical history, clinical (e.g. BP, HbA1C and cholesterol) and anthropometric measures were comparable between both arms at baseline (Table 1).

**Table 1:** Baseline characteristics of study participants by arm

	Intervention (*n* 344)		Control (*n* 271)	
	*n*	%	*n*	%
Age, year				
Mean	34·3		34·7	
sd	9·5		9·3	
Gender, Female	159	46·2	133	49·1
Education, years of schooling				
Mean	7·0		7·1	
sd	3·4		3·5	
Marital status				
Married/married-like	229	66·8	179	66·1
Single	88	25·7	52	19·2
Divorced	18	5·3	37	13·7
Birth country, Mexico	330	95·9	265	97·8
Primary language				
Spanish	255	74·1	207	76·4
Indigenous	78	22·7	57	21
Other	11	3·1	7	2·6
Crop				
Raspberry	179	52·0	156	57·6
Other berry	45	13·1	36	13·3
Not a picker	120	34·9	79	29·2
Job type				
Harvester	221	64·2	190	70·1
Hoop crew	70	20·4	26	9·6
Other	53	15·4	55	20·3
Medical history				
Asthma	6	1·7	3	1·1
High cholesterol	37	10·8	31	11·4
Hypertension	31	9·0	22	8·1
BMI, kg/m^2^				
Mean	28·5		28·3	
sd	4·6		4·7	
Waist, cm				
Mean	91·3		90·4	
sd	10·2		10·2	
HbA1C, %				
Mean	5·6		5·5	
sd	0·6		0·8	
SBP/DBP, mmHg				
Mean	120·4/76·8		120·2/77·1	
sd	13·7/9·0		14·5/9·4	
Total cholesterol, mg/dl				
Mean	175·1		175·7	
sd	39·2		36·1	

DBP, diastolic blood pressure; HbA1C, glycated Hb; SBP, systolic blood pressure.

### Effectiveness of the *PASOS SALUDABLES* intervention

**Fig. 2:**
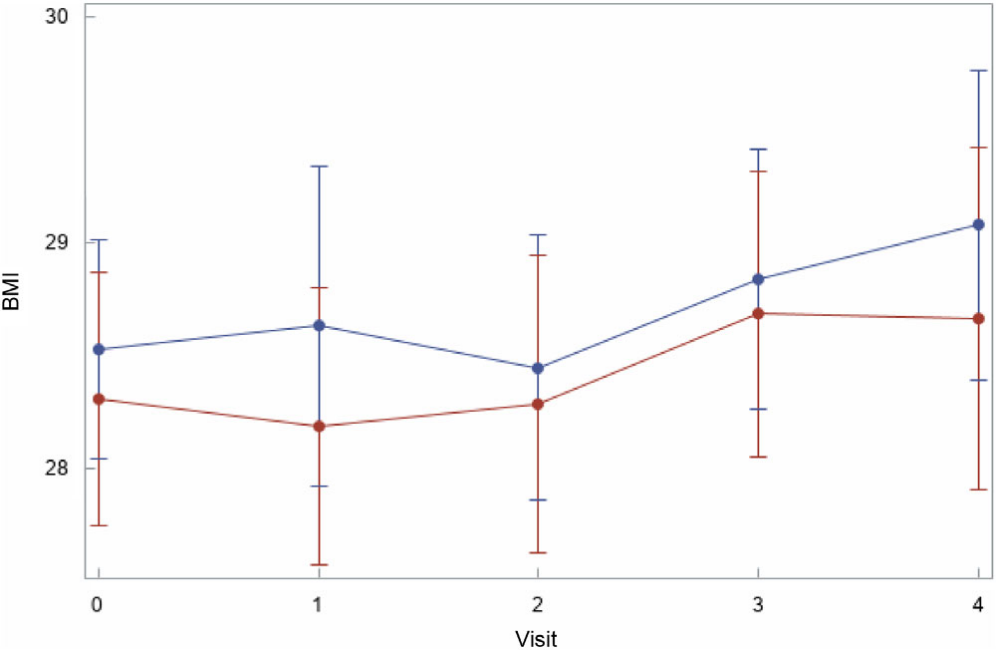
Time variable coded as visit number. Visit 0: baseline; visit 1: end of intervention or 3-month follow-up; visit 2: 6-month follow-up; visit 3: 1-year follow-up; visit 4: 1·5-year follow-up. Blue circles and blue bars denote pointwise unadjusted estimates and 95 % CI for the intervention arm. Red circles and red bars denote pointwise unadjusted estimates and 95 % CI for the control arm

In the analysis of the primary outcome (Table 2), BMI, baseline values did not differ between the intervention and control groups (*P* = 0·72 for treatment at time 0), supporting successful randomisation. Time trend of the control group showed increasing trend of BMI over time (*β* = 0·03 per month, *P* < 0·0001) and that of the intervention group showed attenuated increase of BMI over time, but the difference in trend was not statistically significant (*β* = −0·01 for slope difference, *P* = 0·29). Figure 2 shows the longitudinal data for BMI by group, from baseline (visit 0) to the 1·5 year follow-up (visit 4).

**Table 2:** Effectiveness of the *PASOS SALUDABLES* intervention on primary and secondary outcomes

Factor	*β*	SE	*P*-value
a. Primary outcome: BMI*			
Month	0·03	0·005	<0·0001
Treatment	0·13	0·37	0·72
Month*treatment	−0·01	0·01	0·29
**b. Secondary outcomes**			
Waist circumference†			
Month	0·10	0·02	<0·0001
Treatment	0·51	0·82	0·53
Month*treatment	−0·02	0·02	0·33
HbA1C‡			
Month	0·004	0·001	0·002
Treatment	0·006	0·06	0·91
Month*treatment	0·002	0·002	0·30
Systolic blood pressure§			
Month	0·04	0·03	0·29
Treatment	−0·17	1·08	0·88
Month*treatment	−0·05	0·05	0·27
Total cholesterol‖			
Month	0·43	0·13	0·001
Treatment	−1·22	3·08	0·69
Month*treatment	0·000	0·18	0·99

HbA1C, glycated Hb.

* Sample sizes: 344/271 at visit 0; 263/239 at visit 1; 225/200 at visit 2; 238/220 at visit 3 and 178/152 at visit 4, for intervention/control arm, respectively.

† Sample sizes: 343/270 at visit 0; 262/229 at visit 1; 225/200 at visit 2; 238/216 at visit 3 and 177/154 at visit 4, for intervention/control arm, respectively.

‡ Sample sizes: 342/271 at visit 0; 261/230 at visit 1 and 238/218 at visit 3, for intervention/control arm, respectively.

§ Sample sizes: 344/271 at visit 0; 263/231 at visit 1; 225/200 at visit 2; 238/221 at visit 3 and 177/154 at visit 4, for intervention/control arm, respectively.

‖ Sample sizes: 335/266 at visit 0; 262/229 at visit 1 and 238/218 at visit 3, for intervention/control arm, respectively.

In the secondary outcomes analyses (waist, HbA1C, SBP and total cholesterol), time trend in the control group showed increasing trend in all outcomes, except for SBP (*P* = 0·29). No significantly different trend in any outcome (*P* > 0·27) was observed in the intervention *v*. control groups (Table 2).

Sensitivity analyses results were qualitatively similar to the main analyses. For example, the analysis of the primary outcome (BMI) resulted in *P*-values=0·10–0·29 for the primary parameter (*v*. *P* = 0·29 in Table 2) (see online Supplemental Table 1).

## Discussion

This study aimed to evaluate the effectiveness of *PASOS SALUDABLES,* a workplace-based obesity and diabetes prevention intervention in a Hispanic, farmworker population. Using a cluster randomised controlled trial design, we found that participation in *PASOS SALUDABLES* did not reduce BMI nor other chronic disease indicators (i.e. waist circumference, HbA1C, blood pressure and cholesterol), when it was delivered using a tailgate training format.

The current study findings differ from those previously observed in the pilot intervention where BMI and waist circumference were significantly reduced, particularly among female participants^(14)^. Several differences may explain these contrasting findings. The version of the *PASOS SALUDABLES* intervention evaluated here was adapted in order to meet the shorter format of a meal-break tailgate training. Tailgate trainings are brief, informal training sessions with a small group of workers usually gathered around the tailgate of a truck right in the fields. Switching the delivery of the intervention from employer-run health clinics (as in the pilot) to the worksite fields aimed to reduce participant burden and increase participation, particularly among men. However, this meant reducing the sessions’ duration from 90 to 20 min to be delivered during a meal break, removing the ∼20 min for doing physical activity during the sessions and adding two more sessions to be able to include all the topics. Thus, the intervention dose in this effectiveness study was much lower than that in the pilot. Weight loss interventions with greater dose (i.e. more hours of intervention contact) resulted in greater weight reduction in other populations^(26,27)^. This reduction of direct contact hours from 15 in the pilot to 4 h in this study may explain the contrasting null findings, when compared with the pilot. Another potential explanation may relate to the time during the workday when the intervention was implemented (lunch break), which may have affected the level of participants’ engagement and, consequently, the effectiveness of this educational intervention.

Furthermore, in the pilot study, randomisation was done at the individual level that tend to show larger impact when compared with effectiveness studies allocating geographically defined units (e.g. ranches) to treatment groups^(28)^. Given the agricultural worksite setting, cluster randomisation was the only feasible option to implement the intervention at the actual ranches and the best one to reflect a real-world setting.

On the other hand, this study indicated that implementing a health promotion intervention with farmworkers at their worksite place (i.e. ranches) is feasible. This study enrolled more than six hundred farmworkers and retained 80 % of them through the end of the intervention. Furthermore, this worksite-based approach resulted in higher participation of men (53 %) compared with the pilot study (28 %), which was implemented at community clinics. Hispanic men have experienced a greater increase in obesity prevalence in the past two decades than non-Hispanic White men^(29)^. Thus, the tradeoff between greater reach *v*. lower intervention dose needs to be carefully considered in future implementation of the *PASOS SALUDABLES* intervention. Future implementations may benefit from increasing the number of tailgate sessions to increase back the total amount of contact time (i.e. intervention dose) and conducting the sessions during paid work time, instead of their break time, to increase participants’ engagement and retention.

Several limitations of the study are important to consider. In particular, the inability to blind participants to the intervention, which could have introduced bias. However, the use of an active control group (i.e. control group participants received unrelated education) may have allowed for blinding of participants to the study hypothesis and also accounted for potential treatment effects related to attention received from the study staff^(30)^. Furthermore, the study statistician (data analyst) was blinded to treatment identity. Second, the unexpected changes in work crews in the study ranches (i.e. clusters) may have resulted in cross-contamination. Close communication with ranch managers was maintained to reduce the impact of this labour management change as much as possible. Another limitation is the relatively small number of clusters in the study (six per group). Clustered RCT generally require very large sample size (and many clusters), and although in our study the intra-class correlation for the main outcome (and secondary ones) at the cluster level was essentially zero, it was naturally high within a person. Thus, we cannot rule out lack of power to detect significant differences. Furthermore, as with most longitudinal studies, losses to follow-up occurred (*n* 122, or 20 % by the end of the intervention). We tried to address this by making several attempts to reach participants through the 18 months of follow-up and by including all data available in the repeated measures analysis. Finally, due to missing outcome data for those lost to follow-up and participants with none or very minimum exposure to the intervention, we adopted a less strict intent-to-treat analysis approach (i.e. modified intent-to-treat). Nevertheless, modified intent-to-treat analysis is widely used in longitudinal RCT and does not seem to bias trial results^(31)^ or lead to more favourable results in RCT^(32)^. Throughout our analyses (i.e. main *v*. sensitivity analyses and primary *v*. secondary outcomes), our results are highly robust.

## Conclusions

The *PASOS SALUDABLES* intervention, when it was implemented at the worksite (i.e. fields) during meal breaks using a tailgate format, did not reduce BMI, waist circumference, glycated Hb (a diabetes biomarker), blood pressure or cholesterol among farmworker participants. Despite the potential for greater reach, careful consideration of the trade-offs of delivering a lifestyle intervention as a meal-break tailgate training in the worksite is needed. Nevertheless, this unique study indicated that efforts to engage Latino farmworkers in research and interventions can succeed when research and programming occurs at their worksite.

## Supplementary material

For supplementary material accompanying this paper visit https://doi.org/10.1017/S136898002200129X. click here to view supplementary material

## References

[1] California Department of Food and Agriculture (2020) California Agricultural Statistics Review 2019–2020. Sacramento, CA: California Department of Food and Agriculture.

[2] Martin PL , Hooker B , Akhtar M et al. (2017) How many workers are employed in California agriculture? Calif Agric 71, 30–34.

[3] National Institute for Occupational Safety and Health (2019) National Agricultural Workers Survey (NAWS), Public Data, Fiscal Years (FY) 1989–2018. https://www.cdc.gov/niosh/topics/aginjury/naws/default.html (accessed August 2021).

[4] Kresge L & Eastman C (2010) Increasing Food Security among Agricultural Workers in California’s Salinas Valley. Davis, CA: California Institute for Rural Studies.

[5] Wirth C , Strochlic R & Getz C (2007) Hunger in the Fields: Food Insecurity among Farmworkers in Fresno County. Davis, CA: California Institute for Rural Studies.

[6] Matias S , Marois M & Schenker M (2020) Prevalence and correlates of food insecurity in Latino farm worker households in California’s central valley. Curr Dev Nutr 4, 237–237.

[7] Moore KL , Mercado J , Hill J et al. (2016) Disparities in health insurance coverage and health status among farmworkers, Sonoma County, California, 2013–2014. Prev Chronic Dis 13, E45. 27032988 10.5888/pcd13.150519 PMC4825749

[8] Sadeghi B , Schaefer S , Tseregounis IE et al. (2017) Prevalence and perception of childhood obesity in California’s farmworker communities. J Community Health 42, 377–384. 27734245 10.1007/s10900-016-0266-7

[9] Villarejo D , McCurdy SA , Bade B et al. (2010) The health of California’s immigrant hired farmworkers. Am J Ind Med 53, 387–397. 20191600 10.1002/ajim.20796

[10] Knowler WC , Barrett-Connor E , Fowler SE et al. (2002) Reduction in the incidence of type 2 diabetes with lifestyle intervention or metformin. N Engl J Med 346, 393–403. 11832527 10.1056/NEJMoa012512 PMC1370926

[11] Ruggiero L , Oros S & Choi YK (2011) Community-based translation of the diabetes prevention program’s lifestyle intervention in an underserved Latino population. Diabetes Educ 37, 564–572. 21690435 10.1177/0145721711411107

[12] Ockene IS , Tellez TL , Rosal MC et al. (2012) Outcomes of a Latino community-based intervention for the prevention of diabetes: the Lawrence Latino diabetes prevention project. Am J Public Health 102, 336–342. 22390448 10.2105/AJPH.2011.300357 PMC3483988

[13] Koniak-Griffin D , Brecht ML , Takayanagi S et al. (2015) A community health worker-led lifestyle behavior intervention for Latina (Hispanic) women: feasibility and outcomes of a randomized controlled trial. Int J Nurs Stud 52, 75–87. 25307195 10.1016/j.ijnurstu.2014.09.005 PMC4277872

[14] Mitchell DC , Andrews T & Schenker MB (2015) *Pasos Saludables*: a pilot randomized intervention study to reduce obesity in an immigrant farmworker population. J Occup Environ Med 57, 1039–1046. 26461858 10.1097/JOM.0000000000000535

[15] Fitzgerald S , Kirby A , Murphy A et al. (2016) Obesity, diet quality and absenteeism in a working population. Public Health Nutr 19, 3287–3295. 27230727 10.1017/S1368980016001269 PMC5197930

[16] Trogdon J , Finkelstein EA , Reyes M et al. (2009) A return-on-investment simulation model of workplace obesity interventions. J Occup Environ Med 51, 751–758. 19528833 10.1097/JOM.0b013e3181a86656

[17] Baker KM , Goetzel RZ , Pei X et al. (2008) Using a return-on-investment estimation model to evaluate outcomes from an obesity management worksite health promotion program. J Occup Environ Med 50, 981–990. 18784545 10.1097/JOM.0b013e318184a489

[18] Borelli MR , Riden HE , Bang H et al. (2018) Protocol for a cluster randomized controlled trial to study the effectiveness of an obesity and diabetes intervention (PASOS) in an immigrant farmworker population. BMC Public Health 18, 849. 29986676 10.1186/s12889-018-5560-0 PMC6038353

[19] Ackermann RT , Finch EA , Brizendine E et al. (2008) Translating the diabetes prevention program into the community. The DEPLOY pilot study. Am J Prev Med 35, 357–363. 18779029 10.1016/j.amepre.2008.06.035 PMC2610485

[20] Alcalay R , Alvarado M , Balcazar H et al. (1999) Salud Para Su Corazón: a community-based Latino cardiovascular disease prevention and outreach model. J Community Health 24, 359–379. 10555925 10.1023/a:1018734303968

[21] Secretaría de Salud (2011) Manual de Operación Para las Intervenciones Contra el Sobrepeso y la Obesidad en el Marco del Acuerdo Nacional de Salud Alimentaria (Operations Manual for Interventions to Reduce Overweight and Obesity in the Framework of the National Agreement on Food Security). Mexico: Dirección General de Promoción de la Salud, Centro Nacional de Programas Preventivos y Control de Enfermedades (CENAPRECE).

[22] van Raalten F , Hiemstra YL , Keulen N et al. (2019) Level of agreement of point-of-care and laboratory HbA1c measurements in the preoperative outpatient clinic in non-diabetic patients who are overweight or obese. J Clin Monit Comput 33, 1139–1144. 30659411 10.1007/s10877-019-00255-6 PMC6823319

[23] Rapi S , Bazzini C , Tozzetti C et al. (2009) Point-of-care testing of cholesterol and triglycerides for epidemiologic studies: evaluation of the multicare-in system. Transl Res 153, 71–76. 19138651 10.1016/j.trsl.2008.11.010

[24] Pickering TG , Hall JE , Appel LJ et al. (2005) Recommendations for blood pressure measurement in humans and experimental animals. Hypertension 45, 142–161. 15611362 10.1161/01.HYP.0000150859.47929.8e

[25] Fitzmaurice GM , Laird NM & Ware JH (2011) Applied Longitudinal Analysis, 2nd ed. Hoboken, NJ: John Wiley & Sons.

[26] O’Connor EA , Evans CV , Burda BU et al. (2017) Screening for obesity and intervention for weight management in children and adolescents: evidence report and systematic review for the US preventive services task force. JAMA 317, 2427–2444.2863287310.1001/jama.2017.0332

[27] Perri MG , Limacher MC , von Castel-Roberts K et al. (2014) Comparative effectiveness of three doses of weight-loss counseling: 2-year findings from the rural LITE trial. Obesity 22, 2293–2300. 25376396 10.1002/oby.20832 PMC4225635

[28] Victora CG , Habicht JP & Bryce J (2004) Evidence-based public health: moving beyond randomized trials. Am J Public Health 94, 400–405. 14998803 10.2105/ajph.94.3.400 PMC1448265

[29] Ogden CL , Fryar CD , Martin CB et al. (2020) Trends in obesity prevalence by race and Hispanic origin-1999–2000 to 2017–2018. JAMA 324, 1208–1210. 32857101 10.1001/jama.2020.14590 PMC7455882

[30] Lindquist R , Wyman JF , Talley KM et al. (2007) Design of control-group conditions in clinical trials of behavioral interventions. J Nurs Scholarsh 39, 214–221. 17760793 10.1111/j.1547-5069.2007.00171.x

[31] Dossing A , Tarp S , Furst DE et al. (2016) Modified intention-to-treat analysis did not bias trial results. J Clin Epidemiol 72, 66–74. 26562052 10.1016/j.jclinepi.2015.11.003

[32] Montedori A , Bonacini MI , Casazza G et al. (2011) Modified *v*. standard intention-to-treat reporting: are there differences in methodological quality, sponsorship, and findings in randomized trials? A cross-sectional study. Trials 12, 58. 21356072 10.1186/1745-6215-12-58 PMC3055831

