# Supplementary material for: Effectiveness of a worksite lifestyle intervention to reduce BMI among farmworkers in California: a cluster randomised controlled trial
Source: Public Health Nutr. 2022 May 27;25(9):2651–9. doi: 10.1017/S136898002200129X (PMC9378459; doi:10.1017/S136898002200129X)
Supplement: Supplementary file 1 [file S136898002200129Xsup001.docx]

**Supplementary Material**

**Supplemental Table 1.** Sensitivity analysis of the effectiveness of the *PASOS SALUDABLES* intervention on body mass index (primary outcome)

| **a. With 3-levels nested clustering** | | |
| --- | --- | --- |
| Factor | Beta (standard error) | p-value |
| Month | 0.03 (0.01) | <0.0001 |
| Treatment | 0.13 (0.37) | 0.72 |
| **Month*treatment** | **-0.01 (0.01)** | **0.29** |
|  |  |  |
| **b. Visit number as a categorical variable** | | |
| Factor | Beta (standard error) | p-value |
| Visit #1 | -0.04 (0.09) | 0.68 |
| 2 | 0.01 (0.09) | 0.96 |
| 3 | 0.37 (0.09) | <0.0001 |
| 4 (vs. baseline) | 0.57 (0.10) | <0.0001 |
| Treatment | 0.22 (0.38) | 0.56 |
| Visit #1*treatment | -0.20 (0.12) | 0.11 |
| #2*treatment | -0.30 (0.13) | 0.02 |
| #3*treatment | -0.29 (0.13) | 0.02 |
| #4*treatment | -0.10 (0.14) | 0.50 |
| **Overall test (df=4) for visit*treatment yields p=0.10** | | |
|  | | |
| **c. Excluding participants with HbA1C ≥ 6.5% at baseline** | | |
| Factor | Beta (standard error) | p-value |
| Month | 0.03 (0.01) | <0.0001 |
| Treatment | 0.17 (0.39) | 0.66 |
| **Month*treatment** | **-0.01 (0.01)** | **0.26** |
|  |  |  |
| **d. Gender adjustment** | | |
| Factor | Beta (standard error) | p-value |
| Month | 0.03 (0.01) | <0.0001 |
| Treatment | 0.18 (0.37) | 0.63 |
| **Month*treatment** | **-0.01 (0.01)** | **0.29** |
| Female | 1.49 (0.37) | <0.0001 |
